# Bridging and synergistic effect of the pyrochlore like Bi_2_Zr_2_O_7_ structure with robust CdCuS solid solution for durable photocatalytic removal of the organic pollutants†

**DOI:** 10.1039/d0ra00644k

**Published:** 2020-03-02

**Authors:** Venkatesan Jayaraman, Chinnadurai Ayappan, Baskaran Palanivel, Alagiri Mani

**Affiliations:** Department of Physics and Nanotechnology, SRM Institute of Science and Technology Kattankulathur Kancheepuram 603203 Tamil Nadu India alagirim@srmist.edu.in

## Abstract

Herein, a strong redox ability photocatalyst of CdCuS solid solution composited with pyrochlore like Bi_2_Zr_2_O_7_ has been fabricated by the simple hydrothermal method. The robust CdCuS solid solution materials perform the supporting role to the Bi_2_Zr_2_O_7_ nano materials. The structural, optical, valence and vibrational states of the prepared heterostructure materials were analyzed using various characterization techniques. The photocatalytic activity of the as-synthesized Bi_2_Zr_2_O_7_/CdCuS heterostructure has been verified under direct solar light and ambient conditions. The synthesized Bi_2_Zr_2_O_7_/CdCuS nano combination exhibits a better photocatalytic activity for the removal of methylene blue and 4-nitrophenol organic probe molecules. The heterostructure formation between the samples is confirmed by HRTEM analysis. The improved rate of the photocatalytic reaction of the samples is attributed to the formation of heterostructures at the interface. The close interfacial contact between the two materials discloses the effective charge transfer, which leads to suppressed charge carrier recombination. The enhanced photo catalytic activity of redox-mediator-free-Bi_2_Zr_2_O_7_/CdCuS heterostructure, possibly will be credited to the robust redox ability and the several charge transfer channels in the tight contact. The chief radicals produced in the catalytic reduction reaction have been predicted by the scavenger trapping methods and the results are discussed in detail. The obtained information from this study on Bi_2_Zr_2_O_7_/CdCuS delivers some new visions for the design of active photocatalysts with multiple benefits.

## Abstract 2

Photocatalytic degradation mechanism for CdCuS solid solution supported pyrochlore like Bi_2_Zr_2_O_7_.

## Graphical abstract

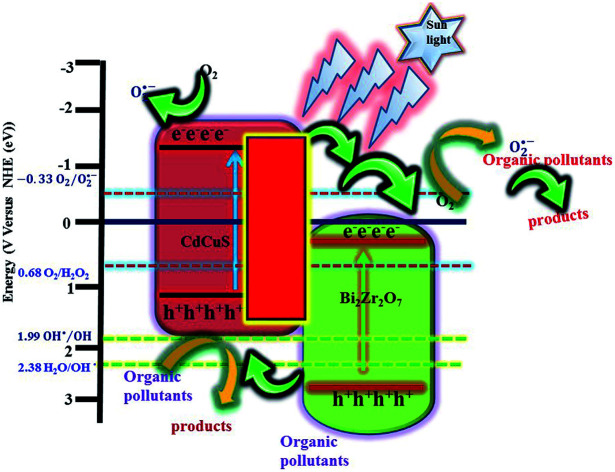

## 1. Introduction

To counter the rising energy demands and increasing pollution, several scholars have given their attention to areas like photocatalytic water splitting and water purification. Water streams have been polluted by industrial effluents like organic dyes, pharmaceutical waste and pesticides. These organic molecules have been considered as harmful to the environment and human beings.^1–3^ In earlier studies, it is reported that 15% of synthetic textile and pharma wastes are abruptly mixed with the main water streams. Colorful dyes and pharmaceutical organic solutions in the water stream will prevent the penetration of light, which totally disturbs the biological processes in the aquatic environment.^4^ Among the industrial dyes, methylene blue (MB) and rhodamine B (RhB) are the main cationic organic molecules used in various industries. Direct disposal of the MB dye will create the following byproducts such as Benzedrine, and methylene. These would-be carcinogenic, which can create various harmful effects for human kind.^5^ It is more difficult to achieve complete removal of such a dyes from the wastewater.^6^ Biological degradation and traditional methods to remove these kind of pollutants are getting attention, though, it is ineffective for decolorization and mineralization of these pollutants.^6,7^

The development of photo catalysts has also relied on well-organized mass transfer that implicates charge carrier migration and reacting radical species transportation. Nowadays, researchers are interested in making semiconductor photo catalysts with hetero structures because of their controlled and effective charge carriers and photon flows. It is believed that the heterostructure catalyst typically signifies a heterojunction, which is from the interface formed between the two or more types of dissimilar solid crystalline materials. On the other hand, the most challenging criteria in both hydrogen and dye degradation by photoactive materials is the active catalytic sites and charge separation using low cost semiconductor materials.^8^ Conventionally, researchers studied metal oxides and sulfides (TiO_2_, ZnO and CdS *etc.*), but unfortunately these materials have certain drawbacks to implicate the materials in industrial applications, such as wide bandgaps, smaller quantum yields, rapid recombination of charge carriers and photo corrosion.^9,10^

Recently bismuth based photocatalysts such as BiVO_4_, Bi_2_WO_6_, Bi_2_O_3_, with different phases have attracted much attraction due to the Bi (6s) orbital O (2p) hybridization which can give a blue-shifted valence band, thus reducing the bandgap of the semiconductors.^11–13^ The materials with A_2_B_2_O_7_ formula belongs to pyrochlore or defect fluorite structure has been frequently used as a catalyst for the removal of organic pollutants effectively. In earlier research works, some reports were found with Bi_2_Zr_2_O_7_ pure pyrochlore as well as defect fluorite and other phase structure.^14–17^ Jyoti Pandey *et al.* reports the detailed study on the pure phase Bi_2_Zr_2_O_7_ pyrochlore for the catalytic removal of the organic pollutants with exceptional band gap (2 eV).^18^ On the other hand, Vaishali Sharma M. *et al.* prepared the same Bi_2_Zr_2_O_7_ defect materials using solution combustion method for the photocatalytic activity for the removal of various organic dyes.^19^

For more efficient and robust photocatalytic organic pollutants the researchers are using the metal sulfides with the visible light responsive bandgap materials. In general, heterostructures photo catalyst constructed with metal sulfides are more effective as well as attractive for the uses due to its relatively visible responsive bandgap compared to metal oxides. Moreover, the metal sulfides act as the photosensitizers which can generate the more number of electrons by absorbing the visible light and easily transfer to the heterostructured materials.^20,21^ Among them, CuS and CdS semiconductor chalcogenides has been considered as a simple and yet powerful photocatalyst for the degradation of organic molecules mixed in the wastewater.^22–24^ These metal sulfides have ideal electronic band structure, more number of catalytic active sites, and sufficient visible region bandgap.^5,21^ The physical and chemical properties of the samples are subjective to its various parameters such as crystalline nature, morphology, compositions, and surface area.^10,25^ Therefore, nano metal chalcogenides are getting much attention due to their unique properties like morphology, high surface-to-volume ratio, low surface penetrance, and low volume density. The metal sulfides act as an efficient catalyst for the degradation of organic pollutants under visible-light due to its narrow band gap. Several techniques have been used for the preparation of pure CuS nanostructures with different morphology and physical properties such as hydrothermal, chemical bath, microwave, wet deposition, and chemical de-alloying.^25^ The above stated approaches falls in the chemical methods category and these are modest and cost effective when compared to other physical ways.^8,26,27^

In this work, Bi_2_Zr_2_O_7_ with robust CdCuS solid solution buoyed heterojunction photo-catalysts was synthesized by simple cost effective hydrothermal method. The various properties of the synthesized heterostructure samples were thoroughly studied. The photocatalytic removal ability of the Bi_2_Zr_2_O_7_ with robust CdCuS solid solution catalysts was measured by the degradation of MB solution under direct sun light illuminations. The efficiency of the materials was analyzed with the mixed dye solution of MB and RhB with the sampling at random time intervals with total time period of 200 min. The novel Bi_2_Zr_2_O_7_ with robust CdCuS solid solution with desirable photocatalytic efficiency for the mixed dyes will be of great importance for practical applications in the degradation of organic dyes. On the other hand 4-nitrophenol photoreduction capability of the material also investigated and reported.

## 2. Materials and methods

### 2.1. Chemicals and reagents

All the chemical, reagents and solvents are purchased from the commercially available analytical grade, and were processed without further purification. Bismuth nitrate pentahydrate (Bi(NO_3_)_3_·5H_2_O), zirconium(iv) oxynitrate hydrate (ZrO(NO_3_)_2_·*x*H_2_O), are from the sigma Aldrich. Ethanol (C_2_H_5_OH)-china AR grade, isopropyl alcohol (CH_3_CHOHCH_3_) SRL-India, ethylenediaminetetraacetic acid (C_10_H_16_N_2_O_8_) SRL India, *p*-benzoquinone (C_6_H_4_O_2_) SRL India, nitric acid (HNO_3_ purity) lobha chemie, and sodium hydroxide (NaOH) Merck India. Cupric nitrate trihydrate (Cu(NO_3_)_2_·3H_2_O) is from Fisher scientific chemicals. Cadmium acetate hydrate ((CH_3_CO_2_)_2_Cd·*x*H_2_O) and thiourea (CH_4_N_2_S) was collected from SRL India.

### 2.2. Synthesis of Bi_2_Zr_2_O_7_ materials

The stoichiometric amount of the Bismuth and Zirconium precursor was used in the synthesis process. The typical preparation for the Bi_2_Zr_2_O_7_ could be as follows, the calculated 5 mM of Bi (NO_3_)_3_·5H_2_O and (ZrO(NO_3_)_2_·*x*H_2_O) solution was dissolved separately in 40 ml of distilled water, subsequently 4 ml of nitric acid was added to the zirconia solution and 1.6 M of 10 ml NaOH solution was added to the bismuth solution. Then, the two precursor solutions were mixed together to form the clear white solution, then it was transferred into the Teflon lined autoclave. After that the solution was subjected into hydrothermal heat treatment at 180 °C for 24 hours, then the precipitate is washed with water and ethanol several times in order to remove the unwanted impurities, At finally it was dried at 60 °C overnight to get powder.

### 2.3. Synthesis of CuS material

For the preparation of pure CuS, 0.1 M of 30 ml thiourea solution was prepared in the distilled water and named as solution A. At the same time, 0.1 M of 30 ml cupric nitrate trihydrate in a distilled water was prepared and named as solution B. Both the solutions were well dispersed for 2 hours by using continuous stirring afterwards the solution A was slowly added with B and sonicated for an hour. The solution was subjected to hydrothermal treatment by 180 °C in the hot air oven with the help of Teflon lined stainless steel autoclave for a duration of 18 hours. Finally the precipitate was thoroughly washed with ethanol and distilled water and sample was dried at 80 °C for 12 hours to get CuS.

### 2.4. Synthesis of pyrochlore Bi_2_Zr_2_O_7_/CdCuS solid solution materials

In a typical process, the prepared Bi_2_Zr_2_O_7_ has been dispersed in CdCuS solid solution in the following way. In a beaker, 0.1 M of 30 ml thiourea was well dispersed in the distilled water. On the other hand, 0.05 M of 30 ml Cadmium acetate hydrate and cupric nitrate trihydrate solutions were prepared separately. After stirring for 2 hours both the solutions was mixed with thiourea solution slowly drop by drop. Then the solution were sonicated in water bath for an hour to get uniform distribution of ions and the weighted amount of 0.2 g of Bi_2_Zr_2_O_7_ samples was added to the above solution, then stirred and sonicated for 1 hour respectively. The obtained solution was kept in the hot air oven in the Teflon lined autoclave with 180 °C for a time period of 18 hours to get the precipitate. Then it was washed with distilled water and ethanol several times to remove unwanted impurities. Finally, the sample was dried at 80 °C for overnight and then grounded for the further characterizations and application. The pure CdCuS sample was prepared by the same synthesis procedure with stoichiometric amount of precursors without addition of Bi_2_Zr_2_O_7_.

### 2.5. Characterization of the sample

The crystalline property of the prepared sample was examined with powder X-ray diffractometer (PANalytical's X'-Pert Pro using (Cu K_α_ radiation *λ* = 1.5406 Å)) instrument. The morphological features of the samples was analyzed with field emission scanning electron microscope (FEG Quanta 250) and HR-transmission electron microscope equipped with energy dispersive X-ray spectroscopy (EDS) instrument ((HR-TEM) with JEOL), Japan. Spectroscopic studies of the samples was identified with the help of the Raman spectra (Horiba-Jobin, LabRAM HR) instrument with 532 nm fixed laser as a excitation wavelength. The surface elemental details of the sample was studied by using the X-ray photoelectron spectrum (XPS-physical electronics instrument) and the received data from X-ray photoelectron spectroscopy (XPS) were corrected by the reference frame of carbon and fitted with Shirley linear background correction. The optical properties of the samples were studied with the help of the UV-Vis diffused reflectance spectro-meter ((UV-Vis-DRS) Agilent technologies Cary series) with BaSO_4_ act as a reference materials. Photoemission (PL) things of the samples were did using suitable excitation wavelength laser.

### 2.6. Photodegradation experiments for MB removal

The degradation of organic MB dye in the existence of prepared samples was investigated at the same time to certify the unique solar radiation level. The photocatalytic degradation experiment was conducted between 11 am to 3 pm during the minimum solar intensity fluctuation. The average solar intensity during the experiment was measured as 40 000 lux by using lux meter. The degradation of pure MB dye, mixed with RhB was also studied in the same environment to estimate the ability to degrade the mixed dyes. For the degradation, 50 mg of catalyst was well dispersed in the volume of 100 ml dye solution. The catalyst containing dye solution was stirred for 30 min to ensure the adsorption and desorption properties. The adsorption amount is ignorable during the dark environment. The catalyst containing dye solution was continuously stirred and exposed to direct solar light. 3 ml of sample was collected at regular time interval to test the concentration changes in the dye solution. The maximum peak for the MB was calculated at 667 nm, for RhB the peak maxima was monitored at 552 nm. The organic dye removal efficiency by photocatalytic activity of the catalyst was measured using following formula,
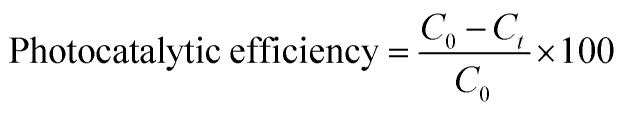
Here, *C*_0_ denotes the initial concentration of the model organic pollutants, and *C*_*t*_ represents the absorption intensity at regular time interval during the photocatalytic experiment under sunlight radiations. The radical trapping experiment was carried out to find the active species in the photoreaction with same experimental procedure with some additives. In the typical experiment process, particular reactive species was trapped with the help of following chemicals such as with 0.5 mol of isopropanol, benzoquinone, ETDA were added during the reaction under sun light radiation and the corresponding results are argued in a detailed manner.

### 2.7. Photo reduction of 4-nitrophenol reaction

The 4-nitrophenol photoreduction reaction was done with above said (Section 2.6) same environment in aqueous solution under solar light. In the reaction, system 50 mg of prepared catalyst was added to the 100 ml of 4-NP solution (10 ppm). Before adding the catalyst, freshly prepared 5 ml of 50 mg NaBH_4_ solution was added. 3 ml of collected solution has been measured with quartz cuvette having a 1.0 cm of length. The absorbance region spectra of the 4-NP solution was measured at regular time intervals in the range of 250–550 nm scanning region.

## 3. Result and discussion

### 3.1. Structural property

**Fig. 1:**
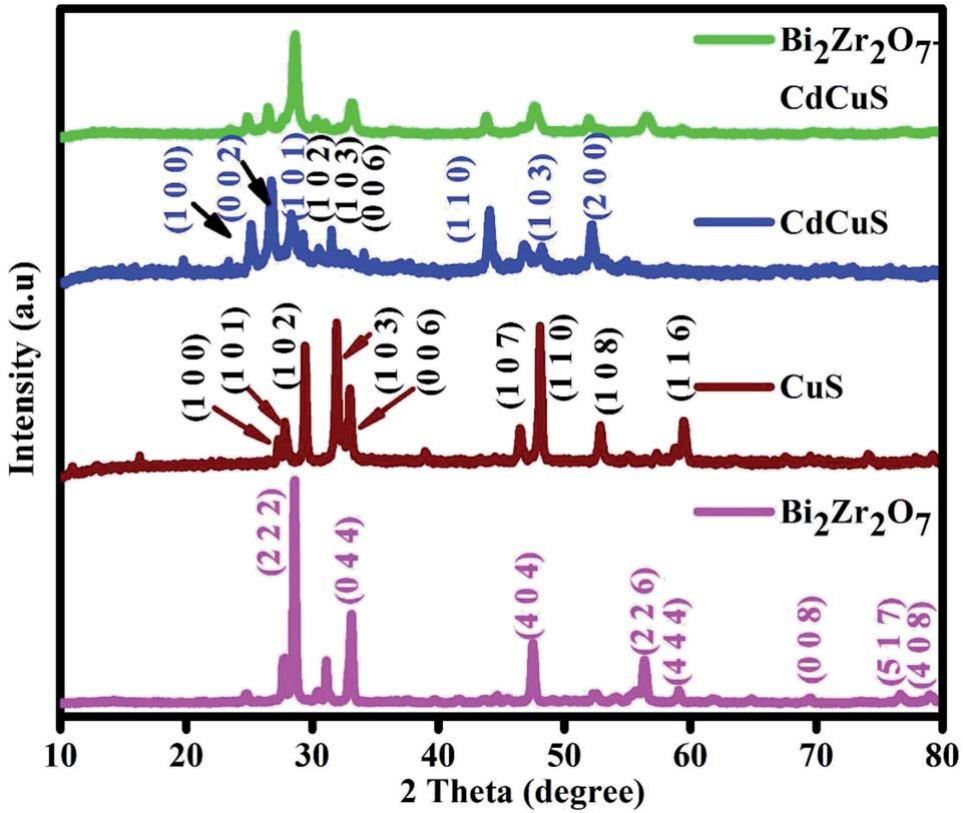
Powder X-ray diffraction of the as-prepared Bi_2_Zr_2_O_7_, CdCuS and CdCuS supported Bi_2_Zr_2_O_7_.

The powder X-ray diffraction (PXRD) tool was used to examine the phase and crystal structure of the synthesized nano catalysts and the respective results are displayed in Fig. 1. All the observed peaks from the PXRD study could be indexed pure cubic pyrochlore like phase with space group *Fd*3̄*m*. The diffraction peaks located at 2 theta values at 28.5, 33.1, 47.4, 56.3, 59.0, 69.2, 76.5, and 78.9 are related to the (2 2 2), (4 0 0), (4 4 0), (6 2 2), (4 4 4), (8 0 0), (6 2 2) and (8 4 0) planes respectively. The above given information is well matched with the previously reported works^18,28–31^ as well as computational work done by the materials project in 2016.^32^ At the same time this reporting Bi_2_Zr_2_O_7_ does not belong to the monoclinic defect fluorite phase which is reported by the other group of researches.^19,33,34^ The characteristic diffraction peaks of the pure CuS was confirmed with the peaks located at 2 theta values at 27.2, 27.8, 29.4, 31.9, 33.0, 38.9, 48.07, and 52.8 are well related to the (1 0 0), (1 0 1), (1 0 2), (1 0 3), (0 0 6), (1 0 5), (1 1 0), (1 0 8) planes of the hexagonal phase with *P*6_3_/*mmc*(194) space group crystal structure with standard JCPDS card No. 65-3556. On the other hand, the major peaks were located approximately around 24.8, 26.4, 28.1, 43.9, 48.1 and 51.8 were related to the hexagonal CdS phase with (1 0 0), (0 0 2), (1 0 1), (1 1 0), (1 0 3) and (2 0 0) respectively with standard JCPDS card No. 80-0006. Similarly, in the prepared CdCuS solid solution the peaks for the secondary hexagonal phase of CuS was slightly observed with slight shift and the comparative details of the pure CdS and CuS with prepared CdCuS solid solution is displayed in Fig. S1.† Form the Fig. S1† it is further confirmed that the prepared samples are not simply physical mixture of the CdS and CuS, but it is formed as solid solution with enhanced chemical and physical properties. In general, physically mixed samples were showed the phases of both structures and their mixed optical properties, but according to the previous reports the solid solution have been displayed CdS single phase with higher or lower angle shift depends on the added secondary phase structure.^35,36^ Obviously, trace amount of impurity phases was observed. It is complicated to control the pure phase solid solution while adding the Cu in Cd_1−*x*_S materials due to its electronegativity and chemical properties. The prepared final sample shows diffraction peaks intensity of both the CdCuS solid solution with pyrochlore like Bi_2_Zr_2_O_7_, no other extra diffraction peaks were found regarding any other impurity phase. The results signifying purity of the final materials. CdCuS solid solution supported Bi_2_Zr_2_O_7_ shows the broad peaks compared to pure Bi_2_Zr_2_O_7_ pyrochlore phase. The utmost intense diffraction peaks of the pure and composite nanoparticles were used to calculate the crystalline size with the help of the Scherer formula. The calculated crystalline size of the Bi_2_Zr_2_O_7_, CuS, CdCuS and Bi_2_Zr_2_O_7_/CdCuS is to be approximately 41, 37, 23, 15 nm respectively, the above result suggesting that the prepared materials comprises of fine nano particles.

### 3.2. Optical properties

**Fig. 2:**
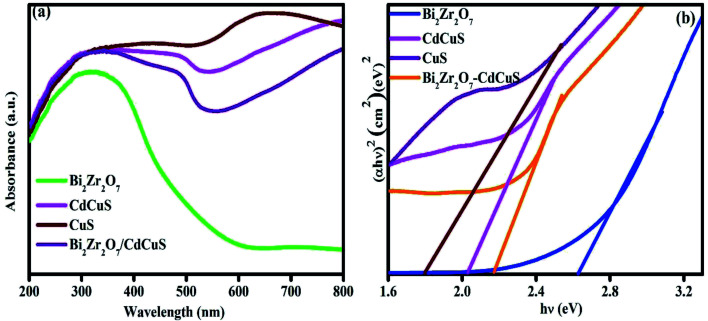
(a) UV-Vis spectra of pure Bi_2_Zr_2_O_7_. CuS, CdCuS, and CdCuS supported Bi_2_Zr_2_O_7_ and (b) corresponding Tauc plots.

To compare and evaluate the optical properties of the samples, the UV-Vis diffuse reflectance spectra (DRS) was taken and the results are shown in Fig. 2a. The pure Bi_2_Zr_2_O_7_ displays an absorption range from 385 to 570 nm, signifying its inadequate photoresponse to visible light region as well as the pure single component has quicker photo generated recombination. On the other hand, the absorption range of CuS and CdCuS shows good response in visible region, when the CdCuS solid solution added to the Bi_2_Zr_2_O_7_ pyrochlore system the absorption edge was significantly shifted towards red shift at visible region. The obtained result from UV spectra suggesting the formed heterojunction between the solid solution and CdCuS will be more active in the visible region. The absorption intensity is also increased when compared to bare samples. The optical band gap of all the prepared semiconductor photocatalysts has been estimated from the Tauc plots by (*αhν*) = *A*(*hν* − *E*_g_)^*n*/2^, in which the symbols *α*, *ν*, and *E*_g_ are the absorption coefficient of semiconductor, light frequency, and band gap energy, respectively, and *A* is the constant. For the prepared samples the band gap was determined from the plots of (*αhν*)^2^*vs. hv* for direct transition. As displayed the Fig. 2b, the optical band gap energies of pristine pyrochlore Bi_2_Zr_2_O_7_, CuS, CdCuS and CdCuS solid solution supported Bi_2_Zr_2_O_7_ samples have been calculated to be 2.6, 1.8, 2.06, and 2.18 eV respectively. The calculated results were accordance with the previous results.^16,37,38^ These results clearly indicates the enhancement of the photo response and their activity of the heterojunction in the prepared composition due to the introduction of CdCuS solid solution into pure pyrochlore phases.

### 3.3. Raman spectra analysis

**Fig. 3:**
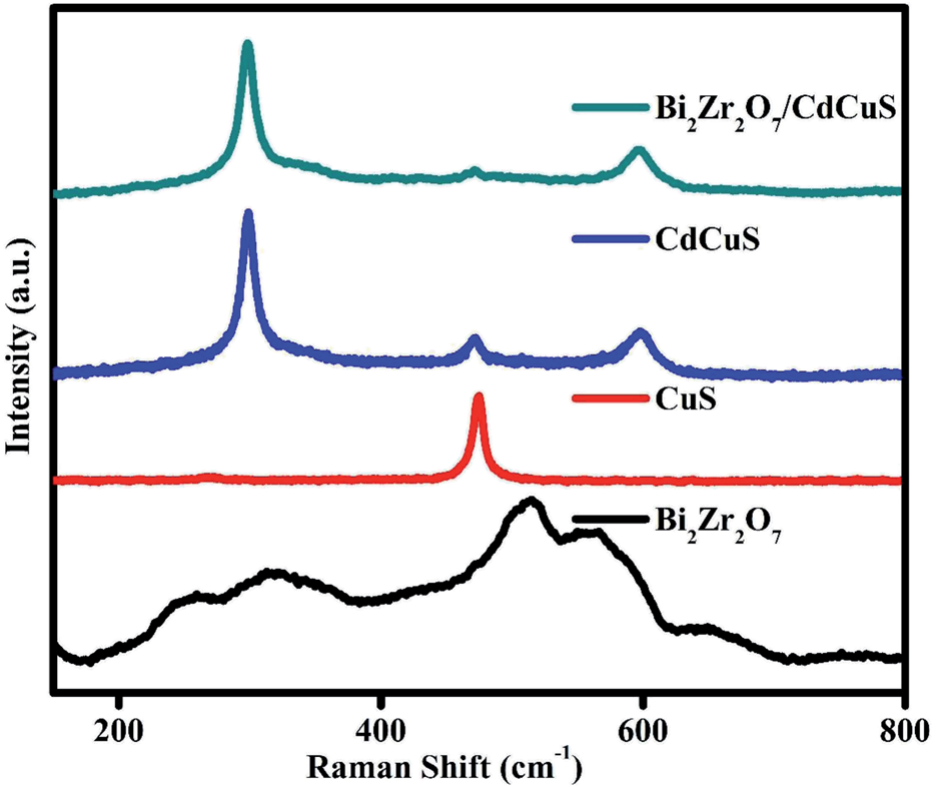
Raman bands of pure Bi_2_Zr_2_O_7_. CuS, CdCuS, and CdCuS/Bi_2_Zr_2_O_7_ nanocomposite.

Raman spectroscopy technique is one of the highly sensitive tool to investigate variation in the crystal structure of the prepared samples. Fig. 3. Shows the Raman spectra of pure Bi_2_Zr_2_O_7_ pyrochlore materials in which six active modes are appeared in region between 200 to 700 cm^−1^, and the modes at 253, 323, 467, 513, 569 and 650 cm^−1^ are signifying that the prepared samples belongs to pyrochlore phase. The observed bands are related to F_2g_(4) at (253, 323, 564 and 650 cm^−1^) E_g_(1) at 467 cm^−1^, and A_1g_(1) at 513 cm^−1^ vibration respectively.^18,30,31^ The broad peak resulting from the combination of first order and second order scattering due to the electron-phonon coupling effects. The hexagonal Covellite CuS primitive unit cell has a total of 36 vibrational modes for CuS system. Among them 14 modes are Raman active, the displayed spectra having the primary sharp peak located at 470 cm^−1^ and one has less intense peak at 265 cm^−1^. The observed CuS Raman peaks at 272 and 470 cm^−1^ are attributed to Cu–S bond vibration and S–S stretching mode of vibration, respectively.^39,40^ The Raman spectra of bare CdCuS solid solution structured material having three distinctive sharp peaks was located at 298, 597 and 470 cm^−1^. The peaks at 298, 597 cm^−1^ are representing the first and second-order LO phonon vibrational modes of CdCuS, respectively and other peak at 470 cm^−1^ is related to the formed secondary phase CuS bands.^41–43^ The peaks of representative hetero structure sample shows Raman bands of Bi_2_Zr_2_O_7_ and CdCuS, the results signifying the formation of good heterojunction.

### 3.4. FE-SEM analysis

**Fig. 4:**
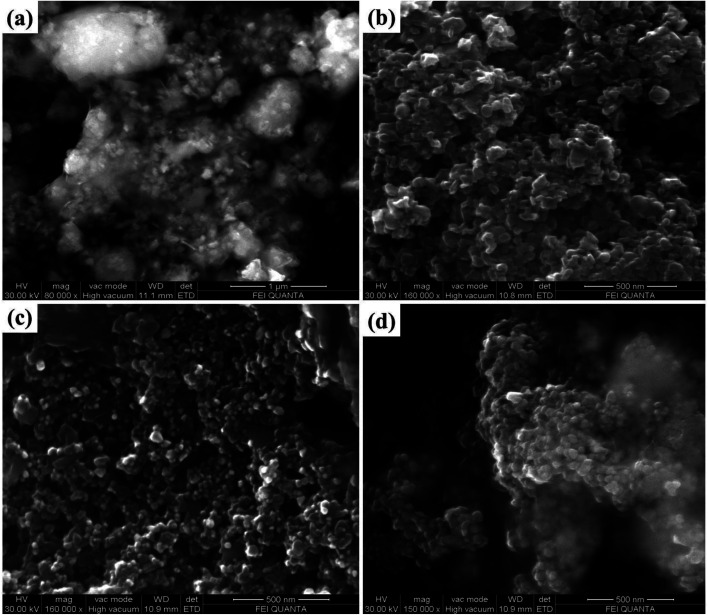
FESEM images of (a) Bi_2_Zr_2_O_7_, (b) CuS (c) CdCuS and (d) CdCuS/Bi_2_Zr_2_O_7_ nanocomposite.

The surface morphological features of the prepared samples was carefully studied by the FESEM analysis technique. Fig. 4, shows that the FESEM images of the Bi_2_Zr_2_O_7_ supported by the CdCuS solid solution together with bare CuS, CdCuS and Bi_2_Zr_2_O_7_ nano materials. From Fig. 4a pure Bi_2_Zr_2_O_7_ materials having the irregular shape and size with nanometer region. The pure CuS in Fig. 4b exhibited mixed plates and spheres like morphology with solid agglomerates. On the other hand, CdCuS (Fig. 4c) solid solution displays the uniform spherical like morphology with minimum agglomeration. After the introduction of CdCuS solid solution into the pure pyrochlore phase Bi_2_Zr_2_O_7_ (Fig. 4d), reveals that the mixed phases of both samples is in uniform distribution. However, there is no distinguishable morphology in the compositions. The average particle size of the Bi_2_Zr_2_O_7_, CuS, CdCuS and CdCuS/Bi_2_Zr_2_O_7_ are to be 69, 60, 37, and 32 nm respectively. The average size of the particle measured from the FESEM is well consistent with the crystalline size measured from the XRD results.

### 3.5. HRTEM analysis of the samples

**Fig. 5:**
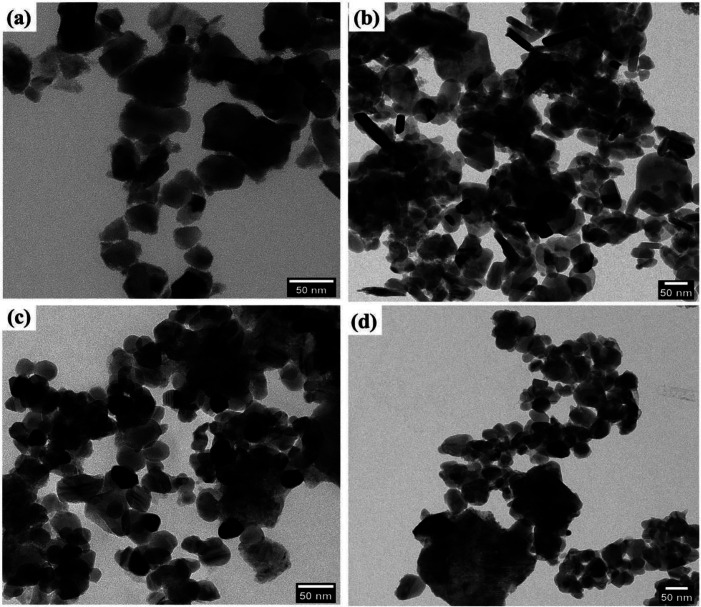
(a–d) TEM images of pure Bi_2_Zr_2_O_7_, CuS, CdCuS and CdCuS/Bi_2_Zr_2_O_7_ nanocomposite.

**Fig. 6:**
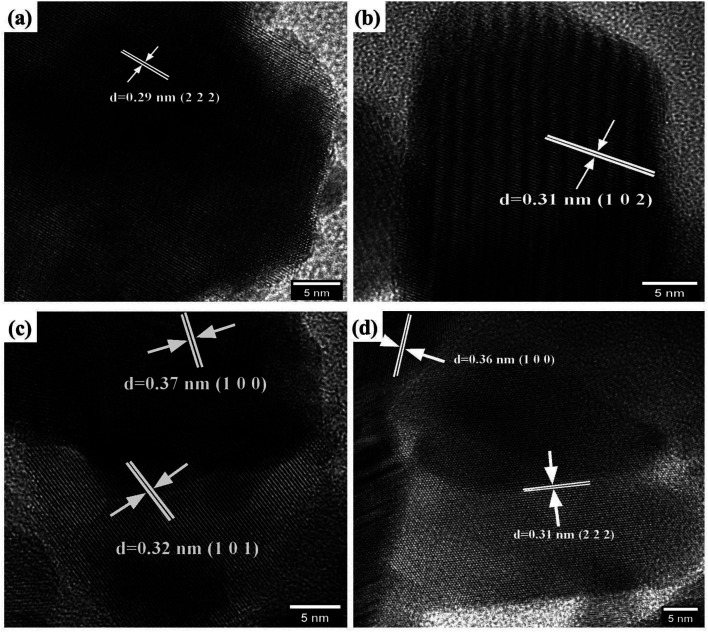
(a–d) HRTEM images of pure Bi_2_Zr_2_O_7_, CuS, CdCuS and CdCuS/Bi_2_Zr_2_O_7_ nanocomposite.

The core morphological features and crystallinity of the samples are additionally explored with HRTEM analysis. Typical overview of the TEM images of the prepared samples is shown in Fig. 5. The prepared pyro-Bi_2_Zr_2_O_7_ shows the non-uniform particle morphology in Fig. 5a. The pure CuS showed the mixed of rods and plates with some degree of aggregations in the morphology (Fig. 5b). On the other hand, the CdCuS solid solution clearly noticed the spherical particle with uniformity in overall places (Fig. 5c). The hetero structured compositions of the materials showed both the pyrochlore Bi_2_Zr_2_O_7_ and CdCuS metal sulfide morphology with less amount of aggregation (Fig. 5d). The HRTEM images of the pure Bi_2_Zr_2_O_7,_ CuS, CdCuS, Bi_2_Zr_2_O_7_ supported by CdCuS is shown in Fig. 6a–d. The lattice spacing of the Bi_2_Zr_2_O_7_ samples showed as 0.29 nm which is well ascribed to the pure phase of pyrochlore (2 2 2) planes. Moreover, the lattice fringes of the CuS shows the spacing of about 0.31 nm for the (1 0 2) plane. The observed lattice spacing of CdCuS solid solution are 0.37 and 0.32 nm which may be belongs to the (1 0 0) and (1 0 1) crystalline facets of CdS hexagonal crystal system respectively. The prepared final composition displays the lattice fringes of all the phase like CdCuS and Bi_2_Zr_2_O_7_ nano materials respectively. Fig. 6d confirms the strong contact between the CdCuS solid solution with pyrochlore cubic structure. Moreover, the intimate contact and crystallinity between constitutes of final samples will provide strong photo activity due to its strong attachment for the degradation of the samples. The pure as well as composition of the prepared samples shows the polycrystalline nature of the samples in the SAED pattern analysis. In addition to that, the SAED pattern from Fig. S2a–d† (Bi_2_Zr_2_O_7_, CuS, CdCuS, Bi_2_Zr_2_O_7_ supported by CdCuS) for the samples further confirms the co-occurrence of CdCuS solid solution and Bi_2_Zr_2_O_7_ planes of cubic phase respectively. The co-existence of the materials once again authorizes the strong heterojunction of the prepared samples, in which the patterns are observed for the pure phases only without the presence of impurity phase. The measured results from HRTEM analyis are well accordance with the above mentioned XRD results.

### 3.6. Elemental analysis

In addition, energy dispersive spectrometer (EDS) analysis was applied to investigate the dispersal of element and their presence in the prepared heterojunction samples and the resultant graph was shown in Fig. S3.† The spectrum shows the exact constitutes in the particular samples without any trace of impurities, in which the calculated elemental composition confirms the constructed samples in the stoichiometric molar ratio. From the EDS results it is further confirmed the formation of solid solution rather than the physical mixture of the both samples in CdCuS materials.

### 3.7. PL analysis and photocurrent measurement

**Fig. 7:**
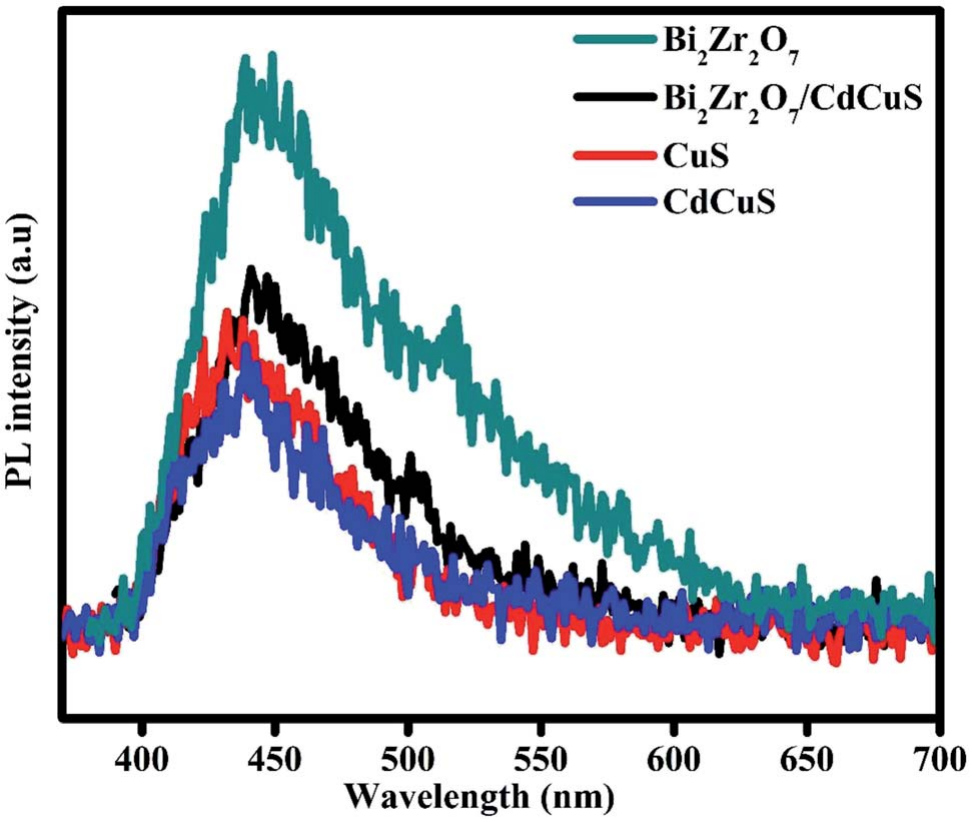
Photoluminescence spectra of Bi_2_Zr_2_O_7_, CuS, CdCuS and CdCuS/Bi_2_Zr_2_O_7_ nanocomposite.

Photoluminescence (PL) spectroscopy is an analytical technique to provision of an efficient charge carrier separation in the prepared CdCuS solid solution supported Bi_2_Zr_2_O_7_ nanocomposites for enhanced photocatalytic activity for the removal of organic pollutant. The emission intensity of PL spectra is due to the rate of recombination of the photo generated charge carriers during the light illuminations in the optical semiconductors. The PL measurement was done with excitation wavelength of 370, 350, 360, and 380 nm for Bi_2_Zr_2_O_7_, CuS, CdCuS and CdCuS/Bi_2_Zr_2_O_7_ respectively, which is found from the UV visible spectra and previous reports.^9,16,27,32,44,45^ As shown in Fig. 7, the PL band of the pure Bi_2_Zr_2_O_7_, CuS and CdCuS and their compositions display a strong emission peak at 439, 431, 448 and 440 nm, corresponding to its band gap and intrinsic properties. According to the Peipei Wan *et al.* the peak around 431 nm in pure CuS could be ascribed to the trap-state emissions of CuS, and connected to copper vacancy in origin.^37,46^ On the other hand, the Kundu Joyjit *et al.* declared that the luminescence property of CuS nanostructures is still not well established.^47^ The CdCuS solid supported Bi_2_Zr_2_O_7_ nanocomposites also display PL emission at the similar region but comparatively much lower intensity than pure Bi_2_Zr_2_O_7_, the results strongly suggests that the lower recombination rate of the material. The PL emission intensity reductions strongly is related to diminished recombination rate, the order of emission intensity follows Bi_2_Zr_2_O_7_ > CdCuS solid solution supported Bi_2_Zr_2_O_7_ > CuS > CdCuS respectively. Obviously the CdCuS showed very lower PL intensity than all the other prepared samples. The reduced PL intensity of CdCuS/Bi_2_Zr_2_O_7_ compared with pure pyrochlore material suggests the efficient charge carrier separation of the prepared heterostructure. The formed heterostructure in turn contribute to the efficient photo activity.

Furthermore, to verify the photoexcited charge carrier separation efficiency during the light illumination the photocurrent measurement was evaluated and the respective results were displayed in Fig. S4.† For the photocurrent measurement 5 mg of prepared catalyst were well dispersed in the Nafian solution and coated in the classy carbon electrode, then it is used for the measurement with three electrode system in the CHI604E electrochemical workstation such as Pt wire, Ag/AgCl and working electrode with 1 M of KOH as a electrolyte. The 500 wt Xenon lamp was used as a light source for the photocurrent experiment. From the Fig. S4.† It is evident that the photoresponse of Bi_2_Zr_2_O_7_/CdCuS was drastically increased compared than pure Bi_2_Zr_2_O_7_ materials, it may be due to the effective charge transfer between the formed heterostructure between the materials. On the other hand, the pure CdCuS showed the very high photoresponse with less stability and the Bi_2_Zr_2_O_7_ is showed very poor response compared than the others. It is further showing the effective utilization of photogenerated charge carrier. From the photoluminescence and photocurrent response it can be concluded that the CdCuS solid solution was acted as a good substrate for the pristine Bi_2_Zr_2_O_7_ pyrochlore like metal oxide substrate.^48^

### 3.8. XPS analysis

**Fig. 8:**
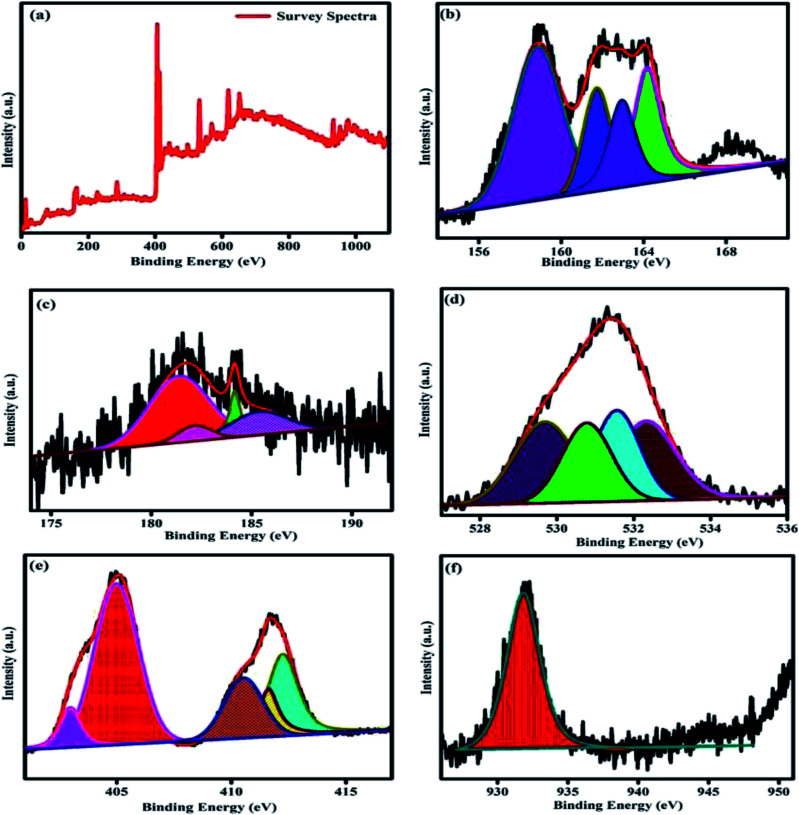
XPS spectra of CdCuS supported Bi_2_Zr_2_O_7_ pyrochlore nanocomposite (a) survey spectrum, (b) Bi, S, (c) Zr, (d) O, (e) Cd and (f) Cu.

XPS analysis was performed to study the valence state of the elements surface composition and synergistic electronic interaction between the two different materials in the prepared samples. The survey scan is displayed in Fig. 8a. The survey spectrum clearly indicates the presence of Bi, Zr, O, Cd, Cu, and S element in the composition and these results are well consistent with the EDS and other characterized tools. The obtained result once again disclose the formation of heterojunction between the two different samples. The high resolution spectra of the Bi 4f S 2p, Zr 3d, O 1s, Cd 3d, and Cu 2p respectively is shown in the Fig. 8b–f. In Fig. 8b displays the high-resolution spectrum of Bi 4f, the two peaks are observed around 158.8 eV and 164.1 eV are related to Bi 4f_7/2_ and Bi 4f_5/2_ of Bi^3+^ oxidation state respectively. The Zr 3d region spectra is shown in Fig. 8c, the spectrum displays four fitted peaks at 181.4, 182.2, 184.1, and 185.5 are ascribed to Zr 3d_5/2_ and Zr 3d_3/2_ respectively. The peak splitting between the Zr 3d_5/3_ and Zr 3d_3/2_ is calculated to be 2.7 eV for 181.4, 184.1 and 3.3 eV for 182.2 and 185.5 eV respectively, which is a characteristic feature of oxidation state of Zr^4+^ ions in the prepared compounds. The O 1s spectrum of the sample is given in Fig. 8d, which can be fitted into four peaks at 529.7, 530.7, 531.5 eV and 532.3 eV. The peak located at 530.7 eV may initiates from the lattice oxygen in the pyrochlore materials. The peak at 529.7 may be related to the oxygen ions in the 8a sites. The other peaks may be due to the oxygen vacancies, defect states and hydroxyl oxygen respectively.^14,33,48^Fig. 8e, displays the high-resolution XPS spectra of Cd 3d, in which the two main peaks are observed at 412.2 eV (Cd 3d_3/2_) and 404.95 eV (Cd 3d_5/2_) indicating the existence of the Cd element in the prepared samples.^44,45^ The high resolution core spectra of the Cu 2p is showed in Fig. 8f. In the particular region of Cu 2p spectra having the peak splitting at 931.8 and 950.79 eV with splitting values of 18.99 eV, may be related to the Cu 2p_3/2_ and Cu 2p_1/2_ states respectively. These values are typical values for Cu^2+^ in the CdCuS solid solution.^39,49^Fig. 8b shows the peak splitting around 163.9 and 162.08 eV binding energy assigned as S 2p_1/2_ and S 2p_3/2_ respective to the prepared CdCuS solid solution.^39,49^ The above result suggests that the information about the valence states of the elements, further confirms the successful formation of the heterojunction between the prepared pyrochlore and CdCuS solid solution.

## 4. Photocatalytic activities

### 4.1. Photocatalytic degradation of MB dye under direct sun light

**Fig. 9:**
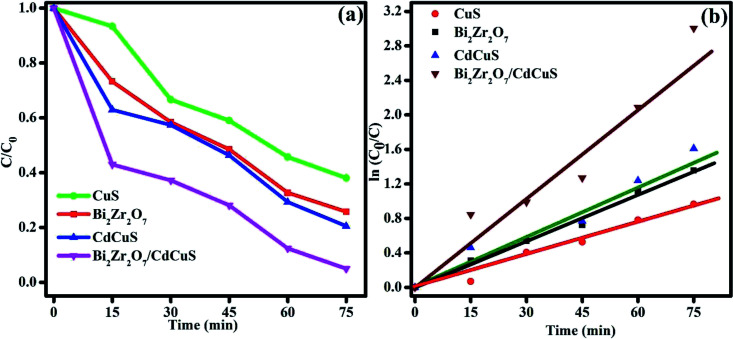
(a) Concentration changes in MB dye solution, (b) time *vs*. kinetics plot.

To assess the photocatalytic removal ability of the organic molecules by the prepared samples, methylene blue was chosen as model pollutants for the photocatalytic degradation process. The photolysis property of the MB solution under sun light without catalyst was performed and the result showed that the photolysis for the MB dye is almost ignorable. The photo degradation efficiency of the pure as well as CdCuS solid solution supported Bi_2_Zr_2_O_7_ materials against MB dye molecules is displayed in Fig. S5,† (a) Bi_2_Zr_2_O_7_, (b) CuS, (c) CdCuS, and (d) Bi_2_Zr_2_O_7_ supported by CdCuS. As a consequence, the prepared pure phase Bi_2_Zr_2_O_7_ pyrochlore supported by CdCuS solid solution exhibits the highest MB degradation efficiency of 96% within 75 min time duration under sunlight irradiation. The prepared CdCuS solid solution almost exhibited 73% of MB degradation efficiency. On the other hand, the initial degradation efficiency of the pure CuS and Bi_2_Zr_2_O_7_ has been found 60 and 74% respectively with the same experimental condition with 75 min time duration, which is too low when compared to the final composition catalyst. The low efficiency of degradation might be from drawbacks of mono phasic catalyst, such as the rapid recombination of photo generated charge carrier and weak band edge potential for the photo redox reactions. The highest efficiency of the materials is due to the effective charge carrier migration between the heterojunction as well as the highly suppressed photo recombination rate of change carriers. It was also realized that the prepared compound exhibits highest activity due to the presence of effective synergistic interaction between nanomaterials and their utilization of charge carriers with suitable redox potentials with normal hydrogen scale. The investigated exceptional photocatalytic degradation efficiency of the heterojunction is 1.6 and 1.2 fold higher than that of pure CuS and Bi_2_Zr_2_O_7_ nano materials respectively. In addition, to know kinetic parameters of the catalytic removal of MB dye, the reaction kinetics was measured. The degradation reaction rates were measured by pseudo first-order kinetics with the following formulaln(*C*_0_/*C*_*t*_) = *k*_*t*_where *C*_0_, *C*, and *k* represents the approximately initial concentration, the concentration at different time interval *t*, and pseudo first-order rate constant, respectively. The concentration changes and the linear relationship between the ln*C*_0_/*C*_*t*_ and time for all the prepared samples are displayed in Fig. 9a and b. The kinetic rate constant for the pure Bi_2_Zr_2_O_7_. CuS, CdCuS and CdCuS supported Bi_2_Zr_2_O_7_ respectively, were calculated and the values were 0.018, 0.012, 0.02 and 0.036 min^−1^ respectively with correlation coefficient (*R*^2^) values to be 0.99, 0.97, 0.96, and 0.94 respectively. From the obtained results, it can be concluded that the proper heterojunction formation between the catalyst will lead to the higher efficiency compared than pure materials, it may be due to the efficient charge carrier utilization with increased materials surface area.

### 4.2. Photocatalytic degradation of mixed RhB and MB dye solution under sunlight

**Fig. 10:**
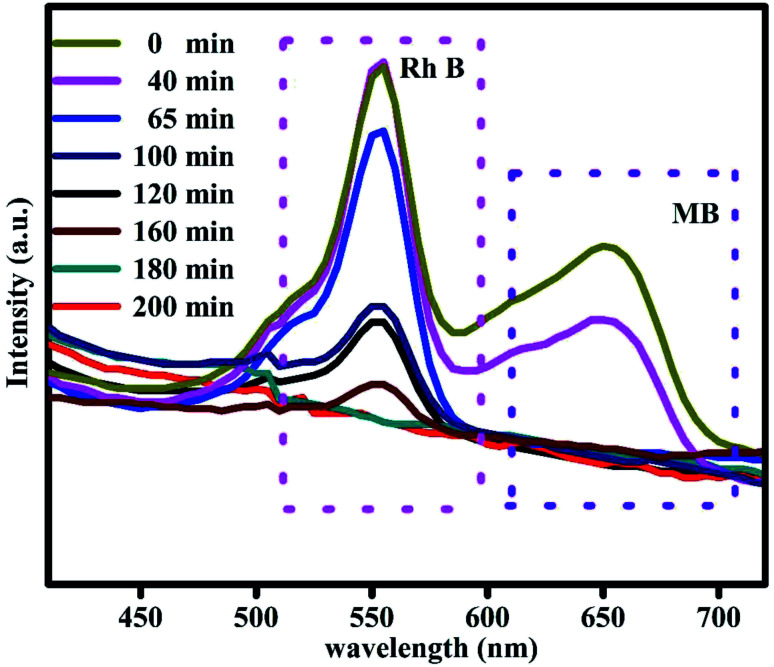
Photodegradation of RhB and MB mixed dye solution.

The photo catalytic efficiency of the Bi_2_Zr_2_O_7_/CdCuS nano structured catalyst for the mixed model pollutants was tested to verify the efficiency for diverse organic pollutant of the catalyst. Methylene blue (MB) and rhodamine B (RhB) are selected as the pope molecules, and an equal amount of these dye solutions was mixed together to form a homogeneous solution. The degradation efficiency of the above mixed dyes were calculated by placing it under direct sunlight with the same procedure which is followed for the degradation of MB solution with 50 mg catalyst content. The adsorption and desorption equilibrium between the catalyst and dye solutions were explored. The absorption intensities of the two different model pollutants were analyzed with random time interval for the total time duration of 200 min. It can be seen from the Fig. 10, the MB solution was degrading quickly when compared to RhB peaks in the mixed solution UV spectra even the concentration of the mixed dyes equal. On the other hand, the RhB dye solution takes more time for degradation due to its more stability when compared to MB dyes. It has been found that the efficiency for RhB peak MB solution were 84 and 90% respectively. From the above results the prepared catalyst gives the best photocatalytic efficiency for the missed dye solution, and it can be useful for the practical applications.

### 4.3. Photo reduction of 4-nitrophenol

After the completion of the several characterization techniques and photo degradation ability for the different color organic dyes of the prepared catalysts, it was found that the prepared catalyst has the enhanced photo response as well as the catalytic activity. Therefore, 50 mg of the pure and different composition catalyst was used to find the photoreduction ability of toxic 10 ppm 4-NP to 4-AP in the presence of NaBH_4_, it was acted as a reductant under direct sunlight with similar environment. Upon the addition of NaBH_4_ reductant into 4-NP, suddenly the color changes was happen from faint yellow to strong yellow with the peak maxima about 400 nm in UV visible spectra. The sudden increase of peak maxima at 400 nm implies the creation of 4-nitrophenolate anions in that solution. The photolysis experiment for the 4-nitrophenol was conducted, the peak at 400 nm remains undiminished even after several hours in the presence of direct sun light. After adding the catalyst, the photoreduction capability of the catalyst was tested.

The photoreduction mechanism of 4-NP was basically classified into two types (1). Hydrogen evolved photocatalysis, (2). The photoreduction with the help of NaBH_4_. The H^+^ ions are generated from the photo catalysis materials when it is having enough water (VB) oxidation potential. The generated H^+^ ions are utilized for the reduction reaction or else it can be converted into hydrogen with the help of e^−^ generated from the CB of the catalyst surfaces. Exceptionally, the materials doesn't have more positive VB, then NaBH_4_ were added into the reaction, which can offer the H^+^ ion and 4-NP was converted into 4-nitrophenolate ions. Afterwards the alkaline nitrophenolate was reduced into 2-aminophenolate ions with less toxic from the electrons generated at the CB band of the reaction system. For this study the NaBH_4_ is essential or else it can lead to complicated reduction pathway.^35,50–52^

**Fig. 11:**
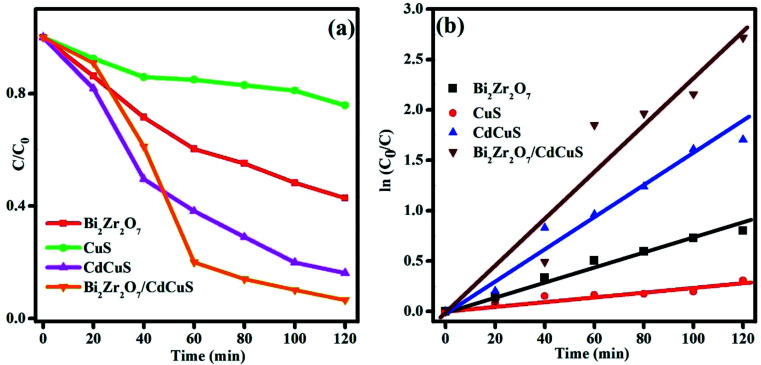
(a) Concentration changes in 4-NP solution, and (b) time *vs*. kinetics plot.

During the photoreduction, 3 ml of the sample was collected and the catalyst was removed by centrifugation and it was used to examine the absorbance by UV-Visible spectrometer. The variation of absorbance spectra of the 4-nitrophenol solution with different catalyst, and fixed time interval was displayed in Fig. S6,† ((a) Bi_2_Zr_2_O_7_, (b) CuS, (c) CdCuS, and (d) Bi_2_Zr_2_O_7_ supported by CdCuS). However, the catalyst was added into the reaction system, the peak maxima at 400 nm was gradually decreasing with regular time interval. A small new peak was emerged around 300 nm due to the formation of less toxic 4-AP colorless solution. Fig. 11a shows the concentration changes of 10 ppm 4-NP solution by various composition of prepared catalyst under sunlight with total time duration of 120 min. It was found that the prepared Bi_2_Zr_2_O_7_/CdCuS composite shows better photoreduction efficiency than the other pure materials. Photoreduction efficiency of the catalyst was calculated using the following formula.Reduction (%) = (1 − *C*/*C*_0_) − 100

The reduction efficiency of the Bi_2_Zr_2_O_7_/CdCuS composite was found to be 96% under sunlight, whereas the pure CuS, Bi_2_Zr_2_O_7_ and CdCuS shows around 26, 55 and 76% respectively with same environment. The rate constant is frequently used as an essential factor to evaluate the activity of the photo catalyst. The rate constant for the given photoreduction reaction was calculated using the pseudo-first order equations. The changes in the concentration with constant time interval and the slops of fitted linear relationship between ln(*C*_0_/*C*_*t*_) and reaction time is given in Fig. 11a and b. The apparent rate constant values of the catalyst was calculated and the corresponding values are given in the Table 1. Obviously, the photoreduction reaction follows the first order kinetics with respect to 4-nitro phenol solution.

**Table 1:** EDS results of the prepared samples

Elements in Bi_2_Zr_2_O_7_	Line type	wt%	wt% sigma	Atomic%
O	K series	4.98	0.40	35.01
Zr	K series	19.92	0.99	24.57
Bi	L series	75.10	1.02	40.42
Total		100.00		100.00

**Element in CuS**				
S	K series	33.55	0.38	50.01
Cu	K series	66.45	0.38	49.99
Total		100.00		100.00

**Elements in CdCuS**				
S	K series	10.68	0.13	22.84
Cu	K series	48.29	0.25	52.12
Cd	L series	41.03	0.27	25.04
Total		100.00		100.00

**Elements in CdCuS/Bi**_**2**_ **Zr**_**2**_ **O**_**7**_				
O	K series	6.93	0.24	26.07
S	K series	10.94	0.25	20.55
Cu	K series	32.90	0.36	31.19
Zr	K series	1.22	0.21	0.81
Cd	L series	30.43	0.44	16.31
Bi	L series	17.58	0.40	5.07
Total		100.00		100.00

From the above discussion it is assessed that the prepared Bi_2_Zr_2_O_7_/CdCuS is more efficient photocatalyst when compared to the pure material. The reason behind the enhanced efficacy is due to the synergistic interaction between the CdCuS solid solution and Bi_2_Zr_2_O_7_ pyrochlore materials^53^ (Table 2).

**Table 2:** Rate constant and efficiency of the 4-nitrophenol reduction

S. no.	Catalyst	Reaction rate (min^−1^)	Efficiency (%)
1	Bi_2_Zr_2_O_7_	0.0072	55
2	CuS	0.0024	26
3	CdCuS	0.0154	76
4	Bi_2_Zr_2_O_7_/CdCuS	0.022	96

### 4.4. Possible photo degradation mechanism and stability analysis

**Fig. 12:**
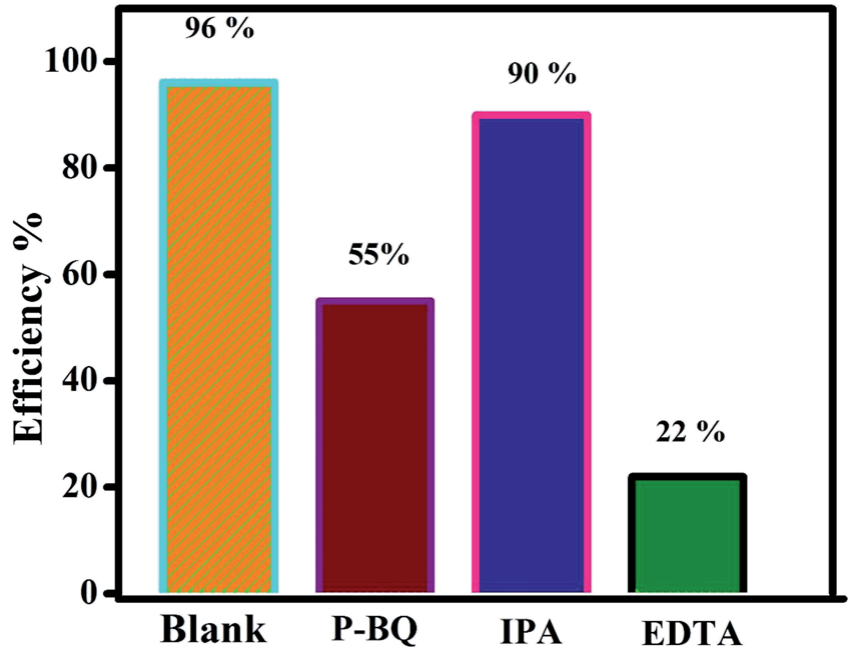
Trapping experiment results.

**Fig. 13:**
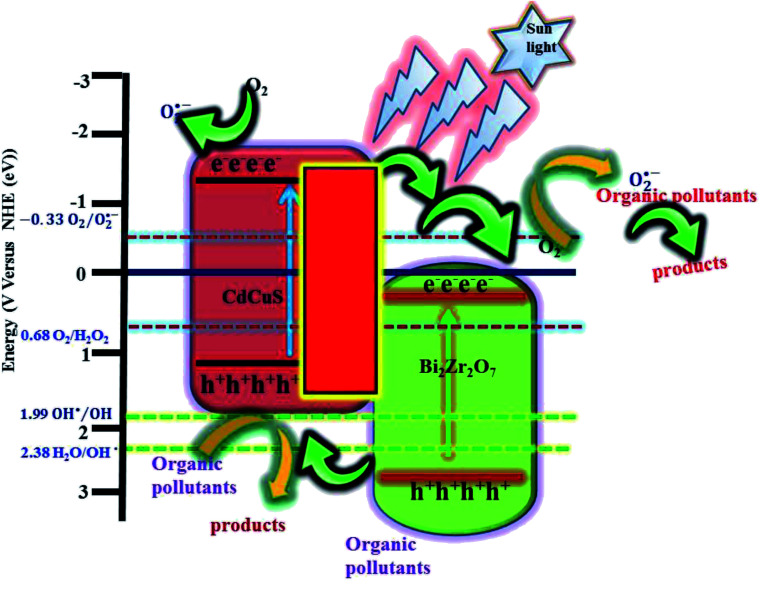
Schematic diagram for the possible photo degradation mechanism.

**Fig. 14:**
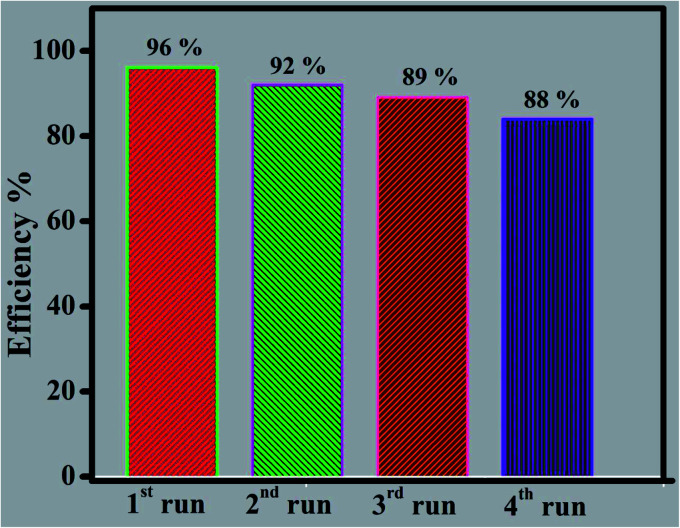
Cyclic stability of catalyst.

It is well known that, different kind of reactive species are generated during the photocatalytic reaction from the H_2_O, surface adsorbed oxygen molecules depend on its band edge potentials. The reactive species are hydroxyl and superoxide radicals, holes, and electrons respectively. In order to find the major reactive species generated during the photocatalytic reaction, a series of trapping experiments were carried with addition of different scavengers. In the present experiment isopropanol, EDTA, and benzoquinone are used as the hydroxyl, holes, and superoxide respectively. As shown in Fig. 12, the addition of all mentioned scavengers in the reaction, the photo degradation efficiency was decreased slightly. However, the addition of (EDTA) act as a h^+^ species hunters in the photocatalytic reaction, the reaction efficiency of the prepared nanocomposite was inhibited, and the degradation efficiency declined from 96 to 22%, signifying that photogenerated holes played a major role in the photodegradation process. However, due to addition of hydroxyl, and superoxide radical's scavengers the efficiency was reduced to 90, and 55% respectively. The valence and conduction band positions for the pure CuS, Bi_2_Zr_2_O_7_ pyrochlore materials has been calculated using the formula as in equation^37,38,54^*E*_CB_ = *χ* − *E*_e_ − 0.5*E*_g_*E*_VB_ = *E*_CB_ + *E*_g_*E*_e_ symbolizes the energy of free electrons with respect to hydrogen standard electrode is about ≈4.5 eV, *E*_g_ represents the optical band gap of the semiconductor photocatalyst. Whereas *χ* is signifies the geometric mean of absolute electronegativity of the atoms. By taking the electronegativity of the CuS and Bi_2_Zr_2_O_7_ are to be 5.29 (ref. 37 and 38) and 6.06 eV,^16^ the band edge calculation was done, accordingly. The calculated conduction and valence band potential positions of the pure CuS and Bi_2_Zr_2_O_7_ are −0.18, 1.76 and 0.26, 2.86 eV respectively. From theory and calculation the photo generated electrons in the CB band of the Bi_2_Zr_2_O_7_ doesn't have the ability to reduce the oxygen into superoxide radicals (O_2_/O_2_˙^−^) −0.33 eV. On the other hand the holes in the valence band can oxidize water into hydroxyl radicals. However, Ranjith R. *et al.* reports that pure CdS has enough CB (−0.39 eV) potentials to produce the superoxide (−0.33 eV) radicals from the reaction.^54^ According to theory, the CB (−0.18 eV) of CuS also does not have the potential to produce the direct superoxide radicals as well as the VB (1.76 eV) also cannot able to produce the hydroxyl radicals. The prepared dye solution with catalyst was placed under the direct solar light after adsorption and desorption equilibrium experiment, in both the Bi_2_Zr_2_O_7_ and CdCuS solid solution semiconductors were excited to generate the electron hole pairs by absorbing the photons from the sunlight. The photo generated electrons were excited to the conduction band from valence band by leaving holes in valence band. The dye degradations and 4-nitrophenol reduction reaction which happens is generally due to heterogeneous catalytic reaction scheme. The organic dye molecules were adsorbed to the surface of the prepared Bi_2_Zr_2_O_7_/CdCuS solid solution photo catalyst and it is oxidized and reduced by the produced radicals in the following way. According to theory, the produced electrons during the photo excitation from the CdCuS solid solutions were possibly moved to the CB of Bi_2_Zr_2_O_7_ pyrochlore materials, simultaneously the electrons from the solid solution also produce the superoxide radicals. On the other hand, the hole from strong potential of Bi_2_Zr_2_O_7_ will move to the weak VB band edge potentials of the CdCuS solid solution. The produced hole from the semiconductors directly react with the organic dye molecules to create the nontoxic byproducts. In addition, the energy produced by light converts water into hydroxyl radicals which directly destroys the model organic molecules in the aqueous solution phase. Here the removal of organic molecules from the water by photodegradation was mainly due to the hole and hydroxyl radicals. The role of superoxide and electron is comparatively ignorable in the reaction system. The schematic explanation of the charge transfer mechanism is given in Fig. 13. In general, the reusability and stability of the photocatalyst is considered as important during the degradation. The reusability of the final composition has been tested for the degradation of MB solution for 4 cycles by reproduction of the catalyst through washing with ethanol and water and dried at 80 °C. On the other hand, the quantity of the loosed samples were included for each cycles to maintain the equal sample contents. The obtained results at the end of four cycles reveals that the catalyst retains the efficiency from 96% to 88% after all the four cycles. The remarkable reusability nature of the prepared catalyst for the MB degradation is reflects the stability of hetero structured materials. The loss of efficiency in the fourth cycle of degradation may be due to its availability of less number of atoms in surface for its reaction. The efficiency of the catalyst for up to four runs and the XRD spectra of the fresh and used catalyst is displayed in Fig. 14, and S7.† The XRD patterns of the used catalyst displays the decreased intensity for CdCuS peaks compared to fresh materials, it may be due to the photo corrosion nature of the metal chalcogenides.

## 5. Conclusion

In conclusion, robust CdCuS solid solution supported pure phase pyrochlore Bi_2_Zr_2_O_7_ has been prepared successfully by simple hydrothermal method and their photocatalytic activity for the diverse organic pollutants is confirmed in the present work. The preparation of CdCuS solid solution on Bi_2_Zr_2_O_7_ and the development of hetero structure between the materials has been proved by serval characterization techniques. The reason behind the efficiency might be due to the interfacial charge transfer between the materials and efficient separation of photo generated charge carriers. The prepared final robust compositions has showed the enhanced photocatalytic activity for the MB, mixed solution of RhB and MB as well as photoreduction 4-nitrophenol. Furthermore, the pyrochlore based metal oxide supported by metal chalcogenides catalyst showed better stability during the photocatalytic activity against MB solution. So the efficiency is retained from 96 to 88% even after four cyclic runs in catalytic reuse. The possible reaction mechanism for the synthesized material has been proposed based on the trapping experiment results.

## Conflicts of interest

There are no conflicts to declare.

## Supplementary Material

RA-010-D0RA00644K-s001
