# Supplementary material for: Bridging and synergistic effect of the pyrochlore like Bi2Zr2O7 structure with robust CdCuS solid solution for durable photocatalytic removal of the organic pollutants
Source: RSC Adv. 2020 Mar 2;10(15):8880–94. doi: 10.1039/d0ra00644k (PMC9050022; doi:10.1039/d0ra00644k)
Supplement: RA-010-D0RA00644K-s001 [file RA-010-D0RA00644K-s001.pdf]

Electronic Supplementary Material (ESI) for RSC Advances.
This journal is © The Royal Society of Chemistry 2020

Supporting information

Bridging and synergistic effect of the pyrochlore like $Bi_2Zr_2O_7$ with robust CdCuS solid solution for durable photocatalytic removal of the organic pollutants

Fig and Table captions

Fig (S1). Comparison of PXRD of the CuS, CdS and CdCuS solid solution

Fig (S2). SAED patterns of the pure of $Bi_2Zr_2O_7$, CuS, CdCuS and CdCuS/$Bi_2Zr_2O_7$ nanocomposite

Fig (S3). EDS spectra of the pure of (a) $Bi_2Zr_2O_7$, (b) CuS ,(c) CdCuS and (d) CdCuS/ $Bi_2Zr_2O_7$ nanocomposite

Fig (S4). Photocurrent measurement of the samples

Fig (S5). Photodegradation of MB dye solution by (a) $Bi_2Zr_2O_7$, (b) CuS , (c) CdCuS ,and (d) $Bi_2Zr_2O_7$ supported by CdCuS

Fig (S6). Photo reduction of 4-NP solution by (a) $Bi_2Zr_2O_7$, (b) CuS , (c)CdCuS ,and (d) $Bi_2Zr_2O_7$ supported by CdCuS

Fig (S7). Stability analysis of catalyst by XRD pattern for before and after the reaction

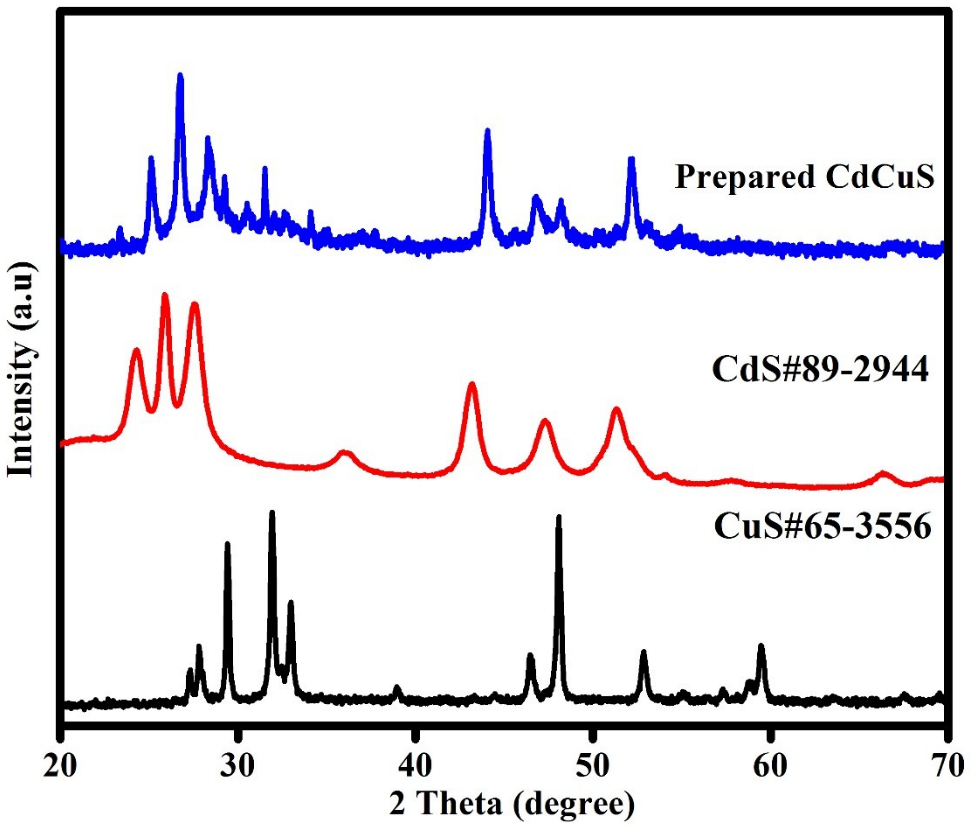

Fig (S1). Comparison of PXRD of the CuS, CdS and CdCuS solid solution

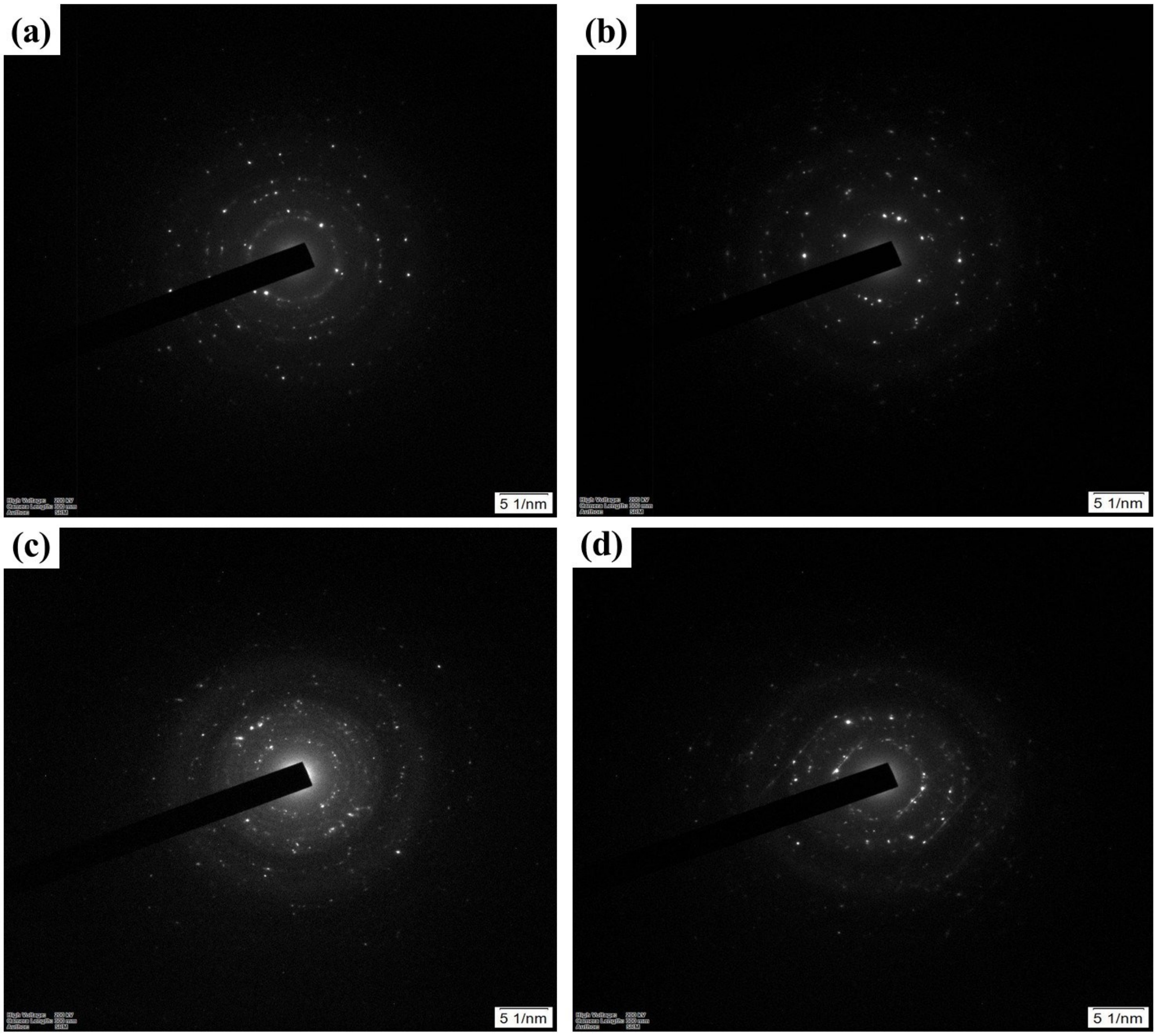

Fig (S2). SAED patterns of the pure of $Bi_2Zr_2O_7$, CuS , CdCuS and CdCuS/ $Bi_2Zr_2O_7$ nanocomposite

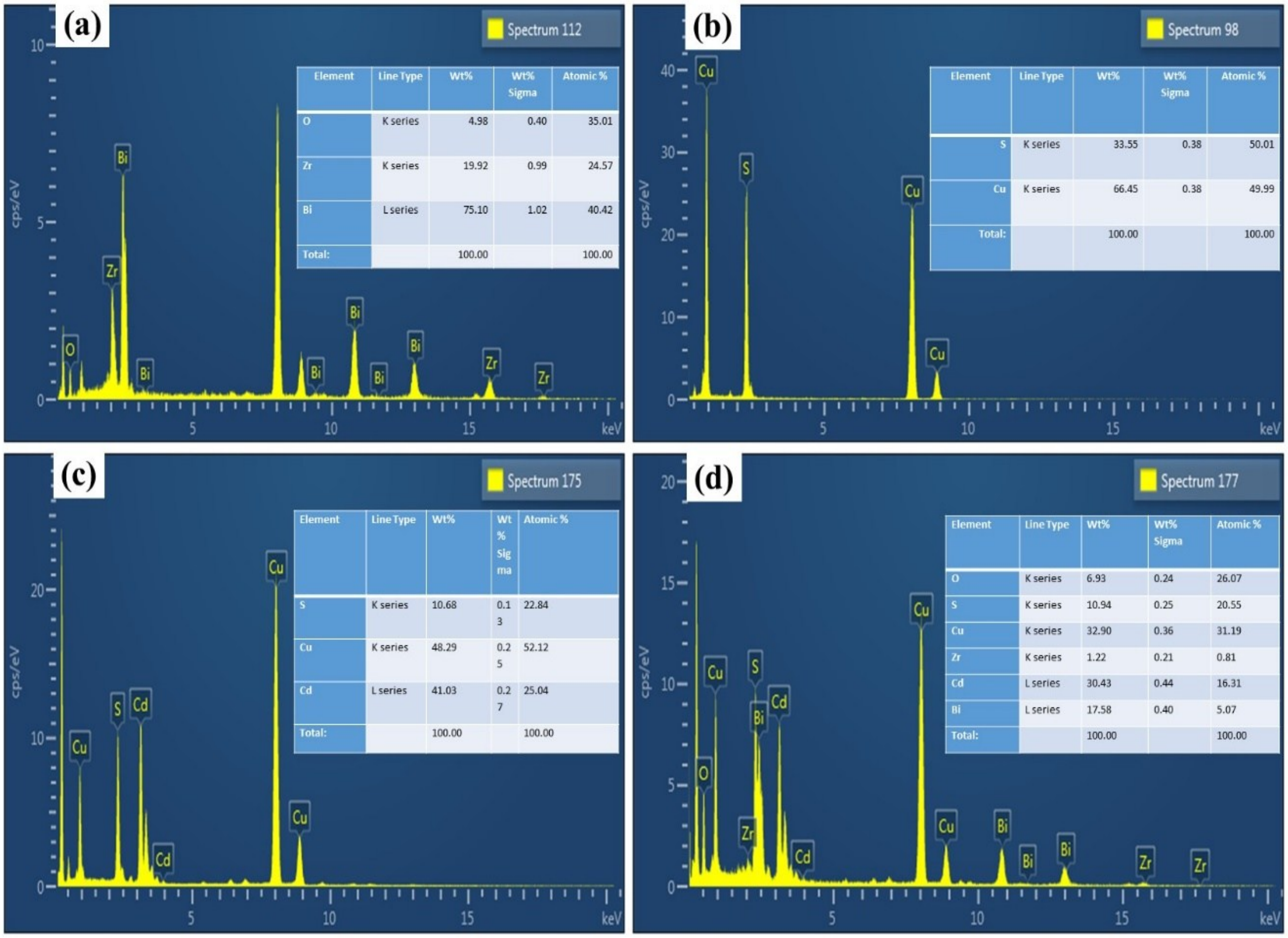

Fig (S3). EDS spectra of the pure of (a) $Bi_2Zr_2O_7$, (b) CuS ,(c) CdCuS and(d) CdCuS/ $Bi_2Zr_2O_7$ nanocomposite

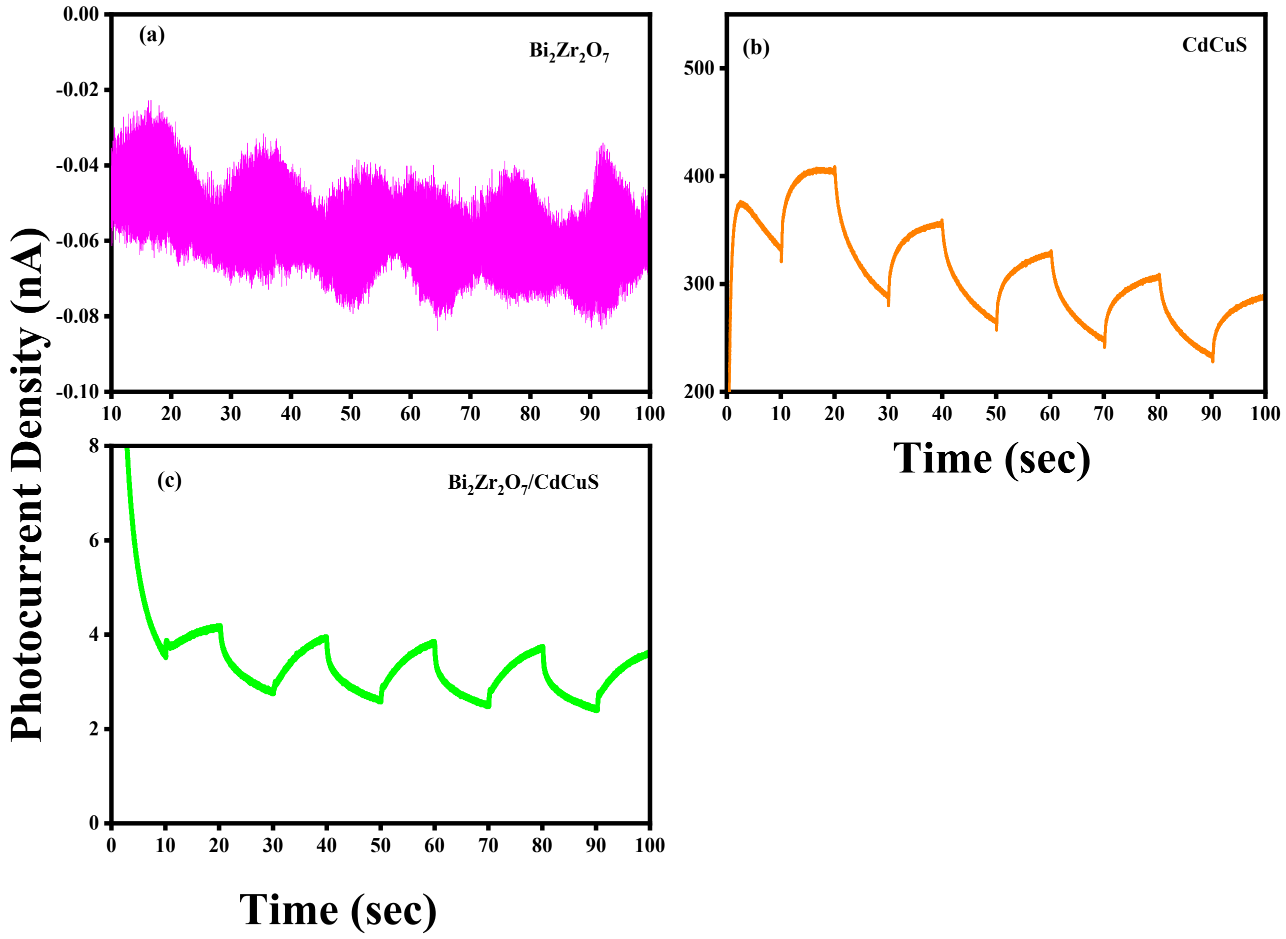

Fig (S4). Photocurrent measurement of the samples

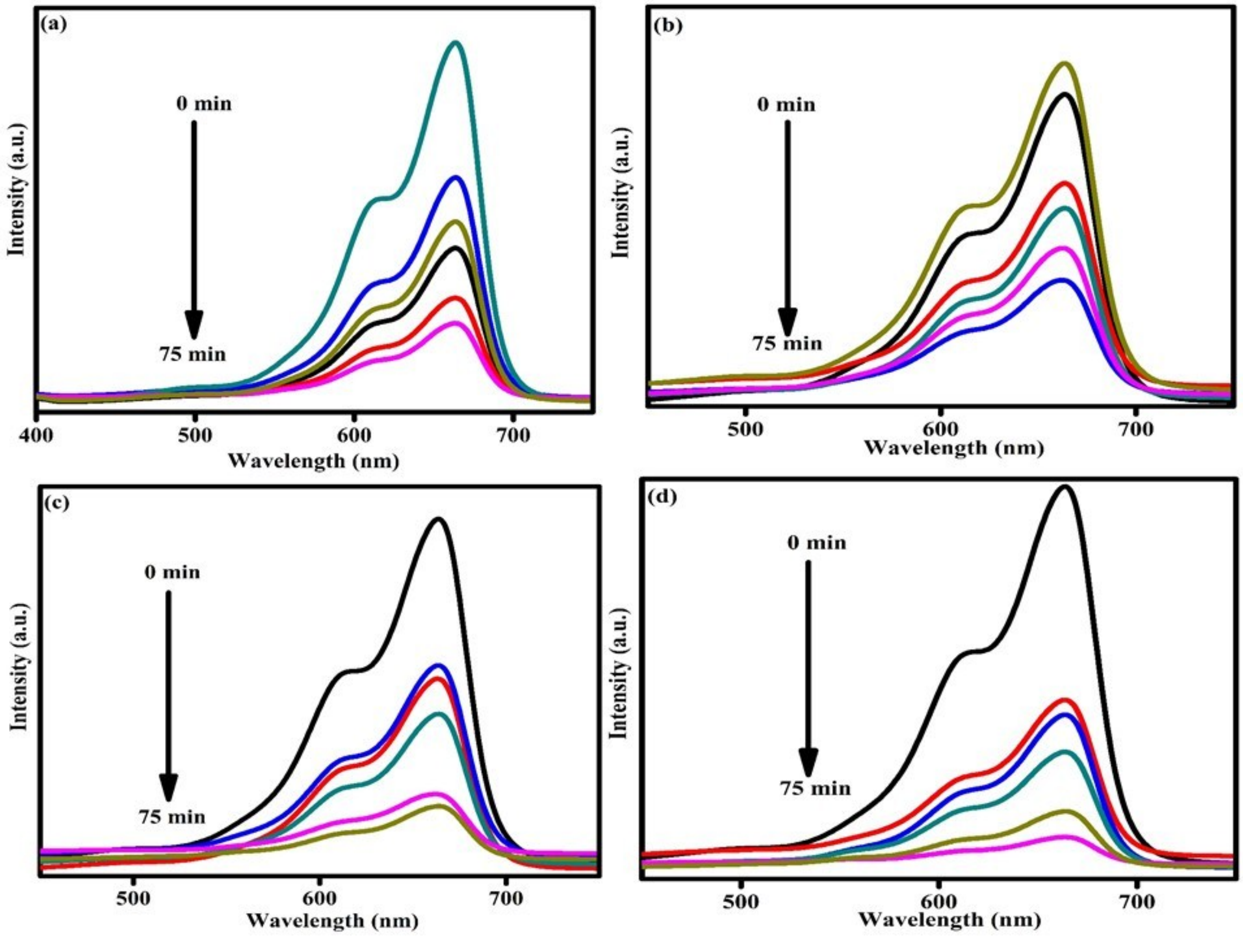

Fig (S5). Photodegradation of MB dye solution by (a) $Bi_2Zr_2O_7$, (b)CuS , (c)CdCuS ,and (d) $Bi_2Zr_2O_7$ supported by CdCuS .

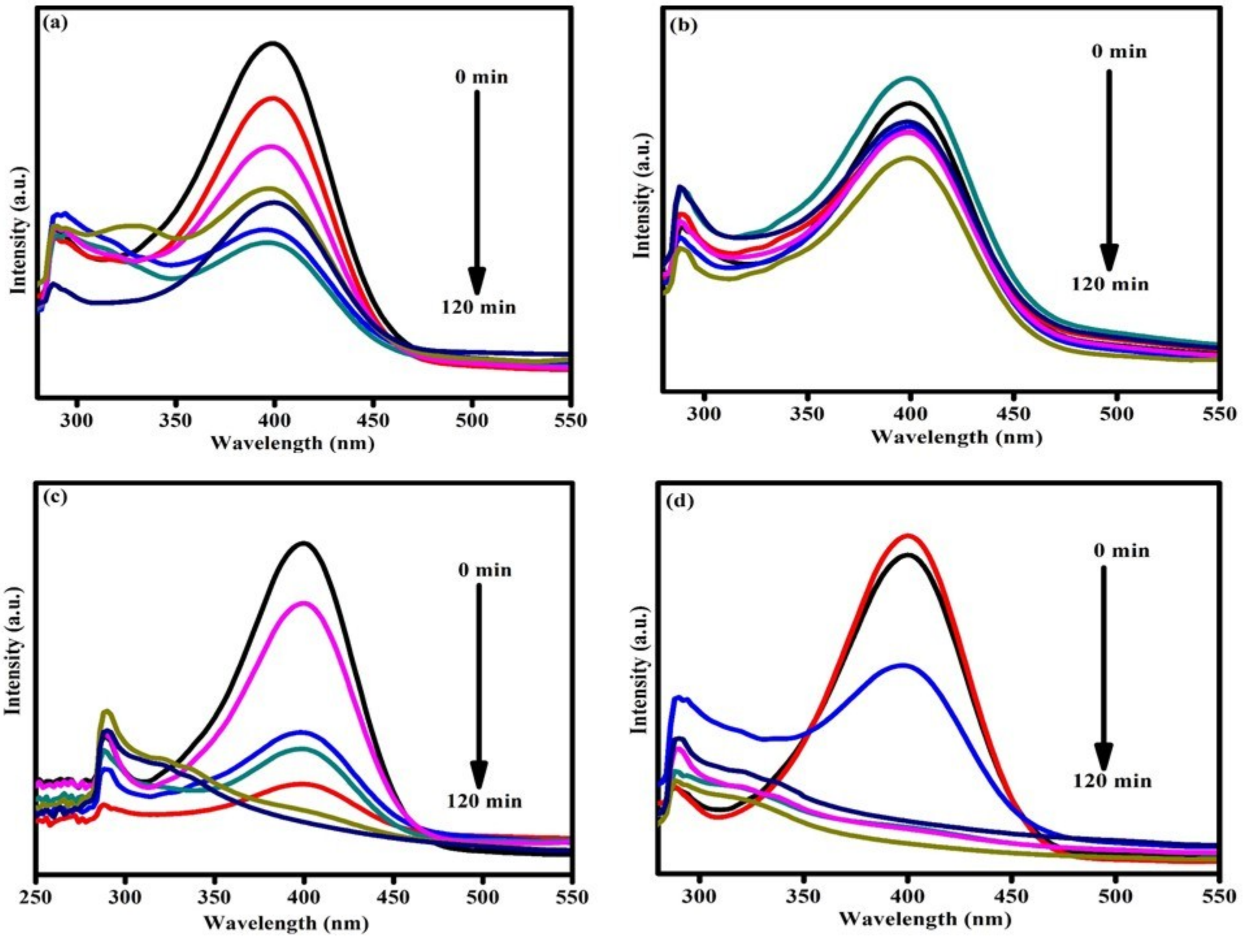

Fig (S6). Photo reduction of 4-NP solution by (a) $Bi_2Zr_2O_7$, (b)CuS , (c)CdCuS ,and (d) $Bi_2Zr_2O_7$ supported by CdCuS .

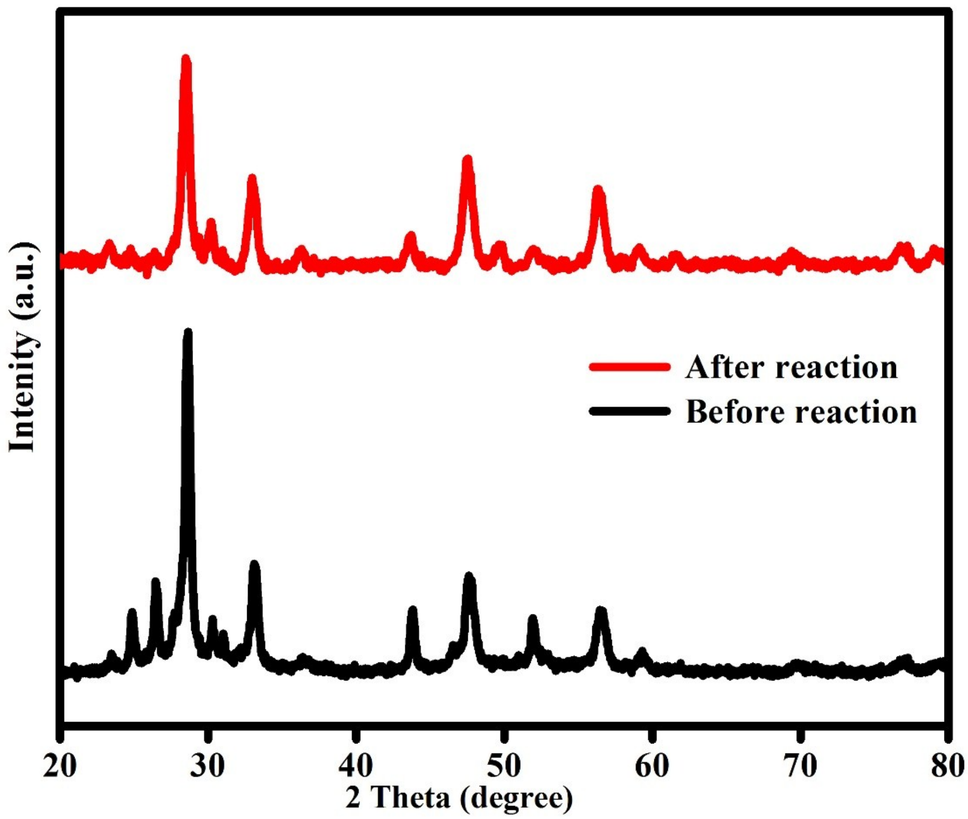

Fig (S7). Stability analysis of catalyst by XRD pattern for before and after the reaction
